# Gender discrepancies and differences in motor and non-motor symptoms, cognition, and psychological outcomes in the treatment of Parkinson’s disease with subthalamic deep brain stimulation

**DOI:** 10.3389/fneur.2023.1257781

**Published:** 2024-01-08

**Authors:** Martijn Hendriks, Ruben Saman Vinke, Dejan Georgiev

**Affiliations:** ^1^Department of Neurology, University Medical Centre Ljubljana, Ljubljana, Slovenia; ^2^Donders Institute for Brain, Cognition and Behaviour, Department of Neurosurgery, Radboud University Medical Centre, Nijmegen, Netherlands; ^3^Laboratory for Artificial Intelligence, Faculty of Computer and Information Science, University of Ljubljana, Ljubljana, Slovenia

**Keywords:** Parkinson’s disease, deep brain stimulation, gender differences, gender discrepancies, cognition and psychological outcomes, motor symptoms, non-motor symptoms

## Abstract

Available data suggest that there may be gender differences in the effect of STN-DBS in the treatment of Parkinson’s disease (PD). The aim of this study was to review data on gender discrepancies and gender differences in clinical outcomes in PD patients treated with deep brain stimulation of the subthalamic nucleus (STN-DBS). Included were original studies that specifically examined gender discrepancies or gender differences in PD patients with STN-DBS. Men receive more DBS than women, for various indications. The decision-making process for DBS in women compared to men is more influenced by personal preferences and external factors. Motor symptoms improve in both genders, but bradykinesia improves more in men. The postoperative reduction of the levodopa equivalent daily dose seems to be more pronounced in men. Men show more cognitive deterioration and less improvement than women after STN-DBS. Women show more depressive symptoms before surgery, but they improve similarly to men. Men show more improvement in impulsivity and less decrease in impulsive behaviour symptoms than women. Anxiety and personality traits remain unchanged in both genders. Voice quality improves more in men and deteriorates less often than in women. Men gain fat-free mass and fat mass, but women only gain fat mass. Regarding sexual function the evidence is inconsistent. More urinary symptoms improve in women than in men. Pain and restless leg syndrome seems to improve more in men. Regarding quality of life, the evidence seems to be inconsistent, and activities of daily living seems to improve in both genders. Better prospective controlled studies, focusing directly on gender differences in PD patients treated with STN-DBS, are needed to better explain gender differences in STN-DBS for PD.

## Introduction

Parkinson’s disease (PD) is a progressive neurodegenerative disease that affects many areas of life. Early cardinal motor symptoms of PD include bradykinesia, rigidity and rest tremor (1). As the disease progresses, other motor symptoms develop, including gait disturbances and postural instability (2). In addition, non-motor symptoms are also common and can greatly affect quality of life and include, for example, autonomic dysfunction, sleep disturbances, cognitive and psychological changes (3). Over the last three decades, deep brain stimulation of the subthalamic nucleus (STN-DBS) has been shown to be an effective treatment for advanced PD (4). The treatment involves the surgical implantation of electrodes into the STN, from where electrical impulses are sent to modulate abnormal neuronal activity. The results of multiple studies indicate a long-term improvement in most motor and non-motor symptoms of the disease (5–7). Emerging evidence indicates that there are important gender differences in the clinical presentation and development of PD (8–10). To illustrate, PD is more prevalent in men (11, 12), in whom the disease seems to start at a younger age (9). On the other hand, tremor-dominant PD is more common in women than in men and women suffer more often from dyskinesias. Similarly, men seem to perform worse on tests of cognitive abilities, but women seem to be more susceptible to depression and anxiety (13). The aim of this review is to investigate gender discrepancies (gender distribution and patient perspective towards STN-DBS) and possible gender differences in clinical outcomes (motor and non-motor symptoms, including cognition and psychological characteristics) in PD patients treated with STN-DBS.

## Materials and methods

**Figure 1:**
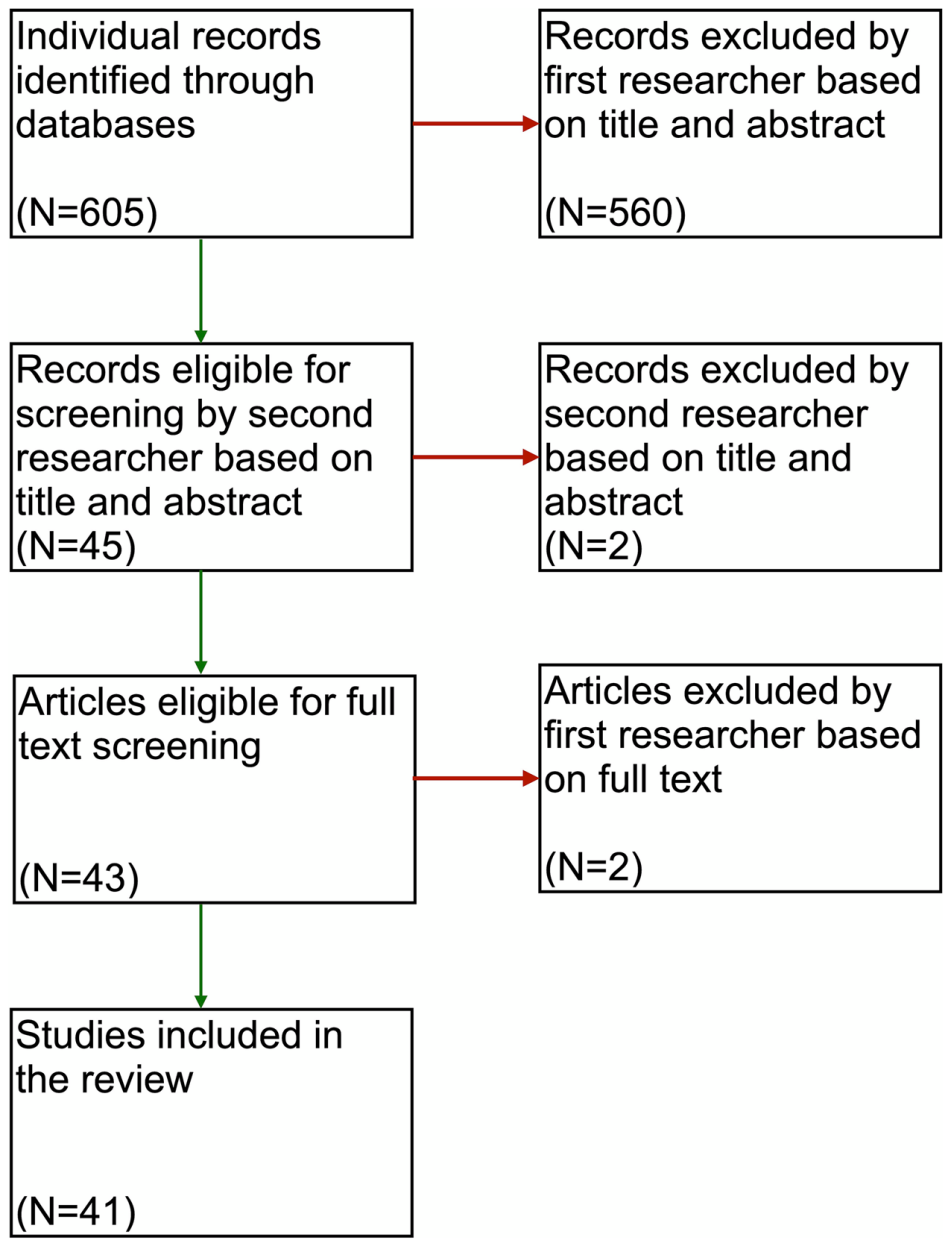
Flowchart of the screening and selection process for articles included in this review.

A systematic search strategy on Pubmed and Web of Science was conducted in May 2023 and included the following search terms and all their synonyms (for the exact search strings please see Supplementary material): Parkinson’s disease, subthalamic deep brain stimulation and gender. The inclusion criteria consisted of primary articles written in English that examined gender differences in PD patients treated with STN-DBS, with outcomes related to gender discrepancies and gender differences in clinical outcomes, including cognitive and psychological characteristics of patients, and other non-motor and motor symptoms. All articles had to be approved by two researchers (MH and DG). Using this systematic search strategy, 605 individual articles were identified. According to the above criteria, 41 individual articles were finally included (Figure 1).

## Results

The results of the analysis are presented in Table 1 and will be discussed in the following sections.

**Table 1:** Studies included in the review.

First author, year	M: N (%)	F: N (%)	Study type	Main objective	O: D	O: M	O: C/P	O: NM	O: Oth	Summary of main gender-related results
Accolla et al. (2007)	22 (58)	16 (42)	Prospective study	To investigate preoperative and postoperative gender differences in PD patients treated with STN-DBS		X			X	Men had more improvement in bradykinesia than women
Andreasi et al. (2022)	71 (66)	36 (33)	Retrospective study	To investigate long term motor effects and gender differences in outcomes in PD patients treated with STN-DBS		X			X	The effect of DBS on LEDD reduction was modulated by sex
Bannier et al. (2009)	15 (68)	7 (32)	Prospective study	To investigate weight gain in PD patients treated with STN-DBS				X		Men gained weight in fat free mass and fat mass, while women only gained weight in fat mass
Bove et al. (2020)	110 (63)	65 (37)	Retrospective study	To investigate dementia after STN-DBS in PD patients			X			Male sex was a predictive factor for dementia after STN-DBS
Castelli et al. (2004)	21 (68)	10 (32)	Prospective study	To investigate the effect of STN-DBS on sexual well-being in PD patients			X	X		Only men improved in satisfaction
Chan et al. (2017)	PD with DBS (targets not specified): 12,366 (68). PD control/overall: 1,276,400 (53)	PD with DBS (targets not specified): 5,946 (32). PD control/overall: 1,131,902 (47)	Retrospective study	To investigate predictive factors for DBS use in PD patients in the United States	X					Male sex was a predictive factor for receiving DBS in PD population
Chandran et al. 2014	32 (63)	19 (37)	Prospective study	To investigate gender differences in preoperative characteristics and post-operative outcomes in PD patients treated with STN-DBS		X	X	X	X	More reduction in LEDD in men than women
Chiou. (2015)	48 (67)	24 (33)	Prospective study	To investigate gender-related predictive factors of outcomes in PD patients treated with STN-DBS		X		X	X	Men with lower LEDD, worse motor scores, worse tremor or better medication response for tremor and rigidity showed better motor improvement. Women with preoperatively worse motor scores, better ADL or better medication response for akinesia showed better motor improvement
Dalrymple et al. (2019)	95 (69) (STN, GPi, Vim)	42 (31) (STN, GPi, Vim)	Retrospective study	To investigate differences of PD patients characteristics based on the primary indication for DBS	X					Men were more likely to undergo DBS for medication refractory tremor than women
Deshpande et al. (2022)	DBS for PD (STN, GPi): 35 (66). DBS for ET (Vim): 27 (52)	DBS for PD (STN, GPi):18 (34). DBS for ET (Vim): 25 (48)	Retrospective study	To investigate a potential gender disparity in the interval from a movement disorder diagnosis to DBS usage in PD patients and ET patients	X				X	At surgery consultation, females had more reduction in UPDRS motor scores with medication than males
Dietrich et al. (2020)	34 (74)	12 (26)	Prospective study	To evaluate effect of STN-DBS on mood and personality with a focus of sex disparities in PD patients		X	X		X	Women showed more improvement in QoL
Erdogan et al. (2020)	20 (51)	19 (49)	Retrospective study	To investigate predictive factors for favourable outcome in PD patients treated with STN-DBS		X				Sex was not a predictive factor for the motor outcome after STN-DBS
Foubert-Samier et al. (2012)	31 (66)	16 (34)	Cross-sectional study	To investigate weight changes after STN-DBS in PD patients				X		Women increased more in BMI than men
Hamberg et al. (2014)	(Targets not specified): 31 (74)	(Targets not specified): 11 (26)	Qualitative cross-sectional study	To investigate the decision-making process prior DBS from PD patient’s perspective and explore gender patterns	X					During the decision-making process prior DBS, women were more represented in the “hesitating and waiting” category than men
Hariz et al. (2013)	31 (63)	18 (37)	Prospective study	To investigate possible gender difference in health quality of life in PD patients treated with STN-DBS		X			X	Women showed improvement in QoL and in more subdomains
Hu et al. (2022)	65 (55)	53 (45)	Retrospective study	To explore the effect and predictive factors of STN-DBS on depression in PD patients			X			Female sex was a predictive factor for the improvement of depression
Jost et al. (2022)	Cross-sectional cohort (STN, GPi, Vim): 214 (68). Longitudinal cohort (STN): 121 (64). PD-controls: 58 (50)	Cross-sectional cohort (STN, GPi, Vim): 102 (32). Longitudinal cohort (STN): 68 (36). PD-controls: 58 (50)	Cross-sectional and prospective controlled study	To investigate gender proportions and preoperative and postoperative gender differences in PD patients treated with STN-DBS	X	X		X	X	Men improved more in bradykinesia than women and only men improved in emotional well-being, mood and apathy, perceptual problems, and hallucinations, while only in women improved in attention and memory
Khazen et al.(2020)	14 (70)	6 (30)	Prospective study	To investigate gender differences in pain in PD patients treated with STN-DBS				X	X	Only men improved significantly on pain scales, of which men improved more than females in musculoskeletal and chronic pain
Kim et al.(2019)	48 (48)	52 (52)	Prospective study	To investigate sex differences in short-term and long-term effects of STN-DBS on clinical outcomes in PD patients		X	X		X	Physical HRQoL improved in more domains in men than in women, which was more prominent at 5 years than 1 year follow up
Kim et al.(2019)	87 (44)	109 (56)	Retrospective study	To investigate the change in functional status after STN-DBS in PD patients					X	Female sex was predictor for functional dependence after STN-DBS
Kübler et al.(2023)	147 (72)	56 (28)	Retrospective study	To evaluate gender-specific post-surgical outcomes in PD patients treated with STN-DBS		X	X	X	X	Only women improved in cognition and only men in depressive symptoms and impulsivity
Lee et al.(2011)	PD STN-DBS: 30 (70). PD-controls/without DBS: 17 (81)	PD STN-DBS 13 (30). PD-controls/without DBS: 4 (19)	Retrospective case-control study	To compare weight changes in STN-DBS PD patients with matched PD patients without STN-DBS				X		Both men and women gained weight
Lee et al.(2008)	STN-DBS: 11 (58). PD-controls/without DBS: 4 (40). Healthy controls: 5 (45)	STN-DBS: 8 (42). PD-controls/without DBS: 6 (60). Healthy controls: 6 (55)	Prospective controlled study	To investigate the effect of STN-DBS on vocal characteristics in PD patients				X		Only men showed better voice characteristics compared to PD controls
Merola et al.(2017)	86 (57)	64 (43)	Retrospective study	To investigate impulse control behaviours after STN-DBS			X			More women developed new-onset ICB than men after STN-DBS
Montaurier et al.(2007)	PD STN-DBS: 17 (71). Healthy controls: 17 (71)	PD STN-DBS: 7 (29). Healthy controls: 7 (29)	Prospective controlled study	To investigate mechanisms of weight gain after STN-DBS in PD patients				X		Women increased in fat mass, while men increase in fat free mass and fat mass
Pedro et al.(2020)	PD STN-DBS: 12 (57). PD-controls/without DBS: 9 (47)	PD STN-DBS: 9 (43). PD-controls/without DBS: 10 (53)	Retrospective study	To investigate the effect of STN-DBS on sexual function in PD patients				X		No effect of STN-DBS on sexual function for both genders
Roediger et al.(2019)	NR	NR	Retrospective study	To investigate the effect of STN-DBS on postural abnormalities in PD patients		X				Male gender was a predictive factor for posture improvement after STN-DBS
Rogers et al.(2011)	10 (45)	12 (55)	Cross-over study	To investigate the effect of STN-DBS on loss chasing behaviour in PD patients			X			The value of losses chased increased more in women than in men with STN-DBS
Romito et al.(2010)	11 (55)	9 (45)	Prospective study	To investigate long-term gender differences in clinical outcomes and disease progression in PD patients treated with STN-DBS		X			X	At 1 year follow-up, men showed more motor improvement than women
Sarac et al.(2020)	8 (67)	4 (33)	Prospective study	To investigate the effect of STN-DBS on voice characteristics in PD patients				X		Only in women some indication for worsening voice quality, but not statistically significant
Shpiner et al.(2019)	DBS-referral: 157 (76). DBS-surgery (targets not specified): 77 (77)	DBS-referral: 50 (24). DBS-surgery (targets not specified): 23 (23)	Retrospective study	To investigate gender disparities in PD patients undergoing DBS	X	X			X	Reasons not to receive DBS: women more likely due to their personal preference, while men were more prone to being lost to follow-up before surgery
Sperens et al.(2017)	PD without DBS: 13 (57)	PD without DBS: 10 (43)	Qualitative cross-sectional study	To investigate knowledge and reasoning about DBS in PD patients	X					Men and women showed no difference between their reasoning about DBS
Su et al.(2017)	11 (48)	12 (52)	Retrospective study	To investigate predictive factors for the predictive value of levodopa responsiveness and predictive factors for STN-DBS outcomes in PD patients		X				Preoperative levodopa responsiveness was a predictor for motor improvement only in women
Tanaka et al.(2015)	PD STN-DBS: 28 (41). PD-controls/without DBS: 15 (38)	PD STN-DBS: 40 (59). PD-controls/without DBS: 25 (63)	Cross-sectional controlled study	To investigate voice characteristics of PD patients treated with STN-DBS				X		Women showed worsening in more voice characteristics after STN-DBS compared to PD controls and improvement in less voice characteristics after switching stimulation off than men
Vinke et al.(2022)	Awake STN-DBS: 25 (83). Asleep STN-DBS: 53 (58)	Awake STN-DBS: 5 (17). Asleep STN-DBS: 38 (42)	Retrospective study	To investigate the change of gender distribution from awake DBS surgical procedure with micro-electrode recording and intraoperative testing to an asleep MRI-guided and CT-verified approach for PD patients treated with STN-DBS	X					More women underwent DBS surgery, after changing from awake to asleep surgery
Wattanabe et al.(2022)	PD with DBS (targets not specified): 49 (66). PD-controls/overall: 2580 (61)	PD with DBS (targets no specified): 25 (34). PD-controls/overall: 1635 (39)	Retrospective study	To characterize the PD population in Hawai and the use of DBS among AA- and NHPI-patients	X					Only males received DBS in NHPI subgroup
Willis et al.(2014)	PD with DBS (targets not specified): 4996 (59). PD control/overall: 331870 (50)	PD with DBS (targets not specified): 3424 (41). PD control/overall: 329987 (50)	Retrospective study	To investigate factors associated with DBS in PD patients	X					Male sex was an independent predictive factor for receiving DBS
Witte et al.(2018)	STN-DBS: 44 (70). GPi-DBS: 44 (68)	STN-DBS: 19 (30). GPi-DBS: 21 (32)	Prospective study	To investigate the effect of STN- and GPi-DBS on lower urinary tract symptoms in advanced PD				X		Both men and women improved in urinary incontinence and frequency
Xie et al.(2011)	PD STN-DBS: 5 (45). Healthy controls: 4 (40)	PD-STN-DBS 6 (55). Healthy controls: 6 (60)	Prospective controlled study	To investigate the effect of STN-DBS on speech in PD patients		X		X		Only within women several significant differences in vowel “i” with different medication and stimulation conditions
Yuan et al.(2023)	55 (61)	35 (39)	Retrospective study	To investigate sex differences on motor and non-motor symptoms and quality of life in PD patients treated with STN-DBS		X	X	X	X	Only men showed motor improvement in med-on conditions and total QoL and more QoL subdomains and men improved more in RLS symptoms
Zong et al.(2019)	PD with DBS (targets not specified): 160 (73). PD-controls/without DBS: 148 (76)	PD with DBS (targets not specified): 60 (27). PD-controls/without DBS: 48 (24)	Prospective study	To investigate effect of DBS on urinary dysfunctions in PD patients				X		Women improved in more urinary outcomes than men

The number of male (M) and female (F) patients, their proportion, study time, main objective of the study, and different outcomes (O): gender discrepancies (O:D), motor outcomes (O:M), cognitive and psychological outcomes (O:C/P), other non-motor outcomes (O:NM), and other outcomes (O:Oth) were extracted and analysed. Summary of the main gender-related findings is also given in the table. AA, Asian Americans; ADL, activities of daily living; BMI, body mass index; ET, essential tremor; GPi, Globus Pallidus internus; (H)QoL, health-related, quality of life; LEDD, levodopa equivalent daily dose; NHPI, Native Hawaiians, and Other Pacific Islanders; NR, not reported; PD, Parkinson’s disease; RLS, restless legs syndrome; STN-DBS, Subthalamic nucleus deep brain stimulation; Vim, ventral intermediate nucleus; UPDRS, unified Parkinson’s disease rating scale. The outcomes addressed in separate papers are marked with “X.”

### Gender discrepancies in PD patients treated with STN-DBS

#### Gender distribution

Two studies showed that male gender was an independent predictive factor for treatment with DBS in PD patients in the United States of America (14, 15). This was later replicated in another study (16). Similarly, the proportion of women in the presurgical evaluation for DBS was lower, but the likelihood of positive approval was higher for women than for men (16). Nevertheless, the proportion of women undergoing DBS was lower in women than in men (16). Another study reported that men were overrepresented in DBS treatment compared to the total number of hospitalizations of patients with PD (17). Dalrymple et al. examined differences based on the primary indication for DBS. They demonstrated that men were more likely to be treated with DBS for medication refractory tremor than women (18). Interestingly, Vinke et al. reported that more women underwent STN-DBS surgery after moving from awake to asleep surgery (19).

#### Gender-specific perspective towards STN-DBS

A study that examined gender disparity from diagnosis to DBS found no gender differences in the interval between diagnosis and surgery or DBS outcomes (20). Interestingly, women had a greater reduction in motor scores before surgery ON medication than men (20). On the other hand, women were more likely to be rejected for DBS because of depression at presurgical evaluation (16) and they were more likely to decide against DBS because of personal preference (21). Men, on the other hand, were more likely not to receive DBS because they had been lost to follow-up in the outpatient clinic before the decision whether to operate has been made (21). One study examined the gender-specific patterns in the decision-making process before DBS (22) and found that while the male decision-making process was mostly characterized by “taking own initiative” and “agreeing when offered”, the female decision-making process was also characterized by “hesitation and waiting”. Women were also more afraid of complications and needed more support from their social environment during the presurgical evaluation (22). In contrast, two other studies found no difference between the arguments for and against DBS (16, 23).

### Gender differences in clinical outcomes in PD patients treated with STN-DBS

#### Motor symptoms

Regarding motor symptoms, a total of 14 studies were analysed, five of which reported better improvement in overall motor scores or subscores in male STN-DBS PD patients and two reported better motor improvement in female STN-DBS PD patients. Most studies reported equal improvement in overall motor function in male and female patients after STN-DBS (16, 24–31). However, three studies reported unequal improvement within bradykinesia subscores (16, 30, 32). Accolla et al. reported equal improvement in total motor scores, but men improved significantly more than women in Unified Parkinson’s Disease Rating Scale (UPDRS) percentage scores for bradykinesia and in absolute hand tapping test scores (30). This is consistent with the findings of Jost et al. who found that total motor scores improved equally in men and women, but bradykinesia improved significantly more in men than in women (16). Interestingly, Yuan et al. demonstrated that both genders improved significantly in “OFF medication-ON stimulation” conditions, but only men improved in the “ON medication-ON stimulation” conditions. This was reported for total scores and most subscores, including the bradykinesia subscore (32).

In a long-term longitudinal study, it was found that the overall motor scores of men improved more than those of women at 1 year follow-up (33). This was related to worse limb akinesia and gait in women than men. However, at 3- and 5 years follow-up, both genders improved equally. Interestingly, only women significantly improved in postural stability. This could be due to women having better overall motor scores, lower score for limb akinesia and lower axial subscore before surgery (33). Another long-term study found equal improvement for total motor scores and subdomains (bradykinesia, axial, rigidity, and tremor) in men and women at 1 year follow-up (25). However, at 5 years follow-up, total motor scores improved equally, but tremor improved significantly only in women (25). In the longest reported follow-up study, they looked at post-surgical motor outcomes in “ON medication-ON stimulation” conditions (34). They found that motor scores and bradykinesia subscores deteriorated in women from 5 years after surgery, but in men from 10 years after surgery. Furthermore, at 1 year follow-up, only men had improvement in rigidity. Women had lower tremor subscores at all times after surgery, but the tremor subscore did not change significantly in neither men nor in women. It is worth mentioning that at baseline women had better total motor scores ON medication, less tremor and less dyskinesias compared to men (34).

In a retrospective study, specifically looking into postural abnormalities, the authors reported that male gender was a predictive factor for upper camptocormia after STN-DBS (35). Two other studies, looking into possible predictive factors for motor improvement, did not find gender as a significant predictive factor for postural abnormalities (31, 36). One of these studies reported that in women with PD preoperative levodopa responsiveness was a better predictor for motor improvement than for male patients (31). Chiou et al. also examined gender-specific predictive factors: men had better motor improvement if they had lower levodopa equivalent daily dose (LEDD) preoperatively, worse motor scores including tremor, or better response to medication of tremor and rigidity (29). On the other hand, women had better motor improvement after surgery if they had worse motor scores preoperatively, better Activities of Daily Living (ADL), or better medication response of akinesia (29).

#### Levodopa equivalent daily dose

Regarding LEDD, a total of eight studies were reviewed, with two reporting a greater LEDD reduction in male patients (24, 37). One 10 years follow-up study reported a significant LEDD reduction only in men but not in women LEDD in whom LEDD returned to values before surgery at 5 years after the surgery (34). However, other studies did not report differences in LEDD reduction between genders (20, 21, 25, 30, 33).

### Cognition and psychological outcomes

#### Cognition

Regarding cognition, a total of seven studies were reviewed, with three reporting improvement or better prognosis of cognition in female patients. Although it is known that STN-DBS does not lead to a decline in general cognitive abilities (38, 39), one study found that male gender was a predictive factor for the development of dementia after STN-DBS (40). In addition, another study found that only women showed significant improvement on cognitive tests after STN-DBS (26). While one study reported improvement in the cognitive domain of the quality of life scale PDQ-39 after STN-DBS (28), another study found no difference in the cognitive domain of the PDQ-39 between genders (16). Other studies report no significant differences in cognition in both men and women after STN-DBS (24, 25, 32).

#### Depression

Regarding depression, a total of seven studies were reviewed (24–27, 32, 41, 42), with one study reporting greater improvement in depression in female patients (41) and one study reporting greater improvement in depression in male patients after STN-DBS (26). Furthermore, a longitudinal study reported that women had more depressive symptoms in all phases of STN-DBS surgery and LEDD was positively correlated with depressive symptoms only in women (27). Although depressive symptoms appeared to decrease more in women than in men, further analysis showed significant modulation by gender, but not for STN-DBS time or the interaction between STN-DBS time and gender (27). Importantly, this and other studies consistently reported that women had more severe depressive symptoms than men before surgery (24, 27, 32, 41). In general, most other studies showed equal improvement in depression in both genders (24, 25, 32, 42).

#### Impulsivity and impulsive disorders

With regard to impulsivity and impulsive disorders, a total of three studies were analysed, one of which found an improvement in impulsivity only in men with PD after STN-DBS (26), and two reporting an increase in impulsivity behaviour in women. Namely, a study examining impulse control behaviours (ICB) after STN-DBS, reported that more women developed new-onset ICB than men, resulting in a higher prevalence of ICB in women (43). In addition, one study examined loss-chasing behaviour as part of gambling behaviour, which is a form of impulse control disorders (ICD) (44), and found that the value of chasing losses increased more in women than men with STN-DBS.

#### Anxiety

Anxiety was measured in two studies, with one study reporting higher anxiety symptoms in women before surgery (32), but neither study found significant improvement in anxiety in men or women after surgery (32, 42).

#### Personality

One study examined the possible personality changes after STN-DBS, in which no significant differences in personality were found before and after STN-DBS in neither men nor women with STN-DBS (27).

### Other non-motor symptoms

Regarding non-motor symptoms in general, a total of four studies were reported, with one study reporting an improvement in certain non-motor symptoms in male or female PD patients with STN-DBS (16). STN-DBS improves the non-motor symptoms as measured by the Non-Motor Symptoms Scale (NMSS) total score (16, 45), regardless of gender, but women improved significantly on the questions on attention and memory and men on the questions on mood and apathy and perceptual problems and hallucinations (16). However, when looking at non-motor symptoms as measured by UPDRS part I, two studies found no significant improvement for neither men nor women after STN-DBS (24, 26).

#### Voice

With regard to the voice, a total of four studies were analysed, two of which reported changes in voice characteristics in men and one of which reported changes in voice characteristics in women with PD after STN-DBS. Namely, Lee et al. demonstrated that only men showed improvement in correlation dimension as a measure for voice quality after STN-DBS compared to patients with PD patients without STN-DBS (46). In addition, two other studies reported that women showed worsening on more voice characteristics (e.g., jitter and shimmer) than men (47, 48) and also improvement on less voice characteristics compared to men OFF stimulation (47), but the results were statistically significant in only one study (47). Interestingly, another study found that only women showed several significant changes in voice characteristics with different medication and stimulation conditions (49).

#### Weight

Regarding weight change after STN-DBS, a total of four studies were analysed, three of which reported gender-specific differences in certain characteristics of weight change after STN-DBS. In general, studies show that men and women endure weight gain after STN-DBS (50–52). Regarding possible gender differences in weight gain, a study showed that after STN-DBS body mass index (BMI) increased more in women than in men (53). Furthermore, in two of these studies both fat-free mass and fat mass increased in men, while only fat mass increased in women (51, 52).

#### Sexual functions

Regarding sexual outcomes, a total of four studies were analysed, one of which reported worse scores in men and one reported improvement of sexual functions in men with PD after STN-DBS. Two studies did not find overall improvement or any gender differences after STN-DBS (42, 54) in sexual functions, with Castelli et al. reporting that only men improved on the dissatisfaction subscore of the reduced version of the Gollombok Rust inventory of sexual satisfaction (GRISS) (42, 55). When considering male patients under 60 years, total improvement of sexual functions was observed on the reduced version of the GRISS (42). On contrary, one study reported that men had worse scores in sexual functions measured by NMSS after STN-DBS (16), Interestingly, Pedro et al. found that age was a predictor of sexual dysfunction in both sexes, but quality of life (QoL) was better in men with erectile dysfunction than in women with sexual dysfunction (54).

#### Urinary dysfunction

Two studies have investigated urinary symptoms and observed improvements in urinary outcomes (urinary incontinence and frequency) for both men and women (56, 57). However, one of these studies, which also included urodynamic tests, specifically reported that women demonstrated improvements in more urinary outcomes, such as residual urine and American Urological Association Symptom Index, than men (57).

#### Pain

One study investigated pain scores showed that only men with PD improved significantly in total pain scores after STN-DBS. Nevertheless, men had more improvement in the subdomains of musculoskeletal and chronic pain (37).

#### Restless legs syndrome

A study investigated restless legs syndrome (RLS) and showed better improvement in men than in women with PD after STN-DBS (32).

### Other outcomes

#### Quality of Life

With regards to QoL and its subdomains, a total of six studies were analysed, three of which reported a better improvement of QoL in men and two in women. Men improve more in QoL (32) and on more subdomains than women (16, 25, 32) as measured by the PDQ-39 (58), PDQ-8 (59), and SF-36 (60). In contrast, Dietrich et al. demonstrated that women showed more improvement in QoL than men (27) and Hariz et al. reported that only women showed improvement in QoL and on more subdomains as measured by the PDQ-39 (28).

#### Activities of daily living

Regarding ADL, a total of nine studies were analysed, with one reporting gender-related differences of ADL after STN-DBS. Namely, after STN-DBS female gender could be a predictive factor for functional dependence (defined as an ADL score below 80%) as measured by the Schwab and England Activities of Daily Living Scale (61, 62). However, most studies report equal significant improvement in ADL in both men and women after STN-DBS (24–26, 28–30, 32, 33).

## Discussion

The aim of this review was to examine gender discrepancies and gender differences in clinical outcomes in PD patients treated with STN-DBS. Evidence from the articles, suggests that there are significant gender discrepancies and differences in clinical outcomes in STN-DBS PD patients. The gender differences in clinical outcomes appear to be more pronounced for cognitive and psychological functions, and other non-motor symptoms than for motor symptoms, although there are also significant gender differences for motor symptoms.

Regarding gender discrepancies, men are more likely than women to receive DBS (14, 15), and their indications differ from women (18). Our observation that men are more likely to receive DBS is in line with a meta-analysis performed by Hariz et al., who also found a global male predominance, except for Asia (63). This could be related to referral bias for surgery in men (13). In addition, the evidence suggests that during the decision-making process for DBS-use, women are more influenced by personal preferences and external factors such as surgical approach and social environment (19, 21, 22). Another possible reason could lie in contraindications for STN-DBS, such as depressive symptoms (16), which are often more severe in women than in men (13).

With regard to gender differences in clinical outcomes, motor symptoms show improvement in both genders (16, 24–31), even in long-term follow-up (25). Men may exhibit more improvement in bradykinesia subscores (16, 30). Interestingly, within the general PD population gender differences are seen in some motor symptoms, such as more dyskinesias and tremor-dominant PD in women (13, 64, 65), but not in bradykinesia. Therefore, a possible gender-specific difference in the bradykinesia response to STN-DBS is not clear and requires further research. LEDD reduction seems to be more frequent in men than women (24). A possible explanation could be that women are set at lower LEDD values before surgery (13), which could explain less reduction. However, these pre-surgical lower LEDD values in combination with less improvement in women was only seen in one study (24). Regarding cognition, the effect of DBS seems to be uncertain, but men may be more susceptible to cognitive decline (40), while women show more frequent improvement in cognitive functioning (26). In general, women seem to outperform men in cognitive tests (13), but this does not necessarily explain a possible gender-specific difference in improvement after STN-DBS. Prior to surgery, women exhibit more severe depressive symptoms (24, 27, 32, 41), which is also observed in the general PD population (13). However, there is evidence of general improvement of depression in both genders after STN-DBS (24, 32). Regarding impulsivity, only men showed improvement in impulsivity scores (26), while women experienced worsening in various impulsivity domains (43, 44). This could be explained by the fact that men with PD are more likely to have an ICD (13, 65) and therefore are more likely to show improvement in these symptoms. Another explanation would be that LEDD decreases more often in men after STN-DBS (24), which could be associate with improvement in ICD (66). Even though some studies suggest possible personality changes after STN-DBS (67), personality traits or anxiety do not appear to change in both genders (27, 32, 42). Regarding other non-motor symptoms, the evidence is not clear as to whether there is an overall improvement (24, 26). Nevertheless, men tend to demonstrate improvement in voice quality (46), while women are more susceptible to voice deterioration (47). In general, STN-DBS seems to lead to deterioration of speech and voice quality (68), and any potential gender differences could maybe be explained by sex-specific anatomical differences (47). Men also show weight gain in fat free mass and fat mass (51, 52), while women only gain weight in fat mass (51, 52). This weight gain for both seems to be explained by a decreased energy expenditure, but unchanged energy intake after surgery (53), while the gender-specific body composition change seems to be explained by a general hormonal difference and response between men and women, known from other fields of research (69). Regarding sexual functions, the results of relevant studies are inconsistent, which leads to the conclusion that there is no clear evidence of improvement in sexual function for both genders after STN-DBS (42, 54). Urinary symptoms improve in both men and women (56), with women seeming to experience a broader range of benefits on urinary symptoms (57). Despite the limited amount of relevant studies, only men appear to improve in pain scores (37) and show improvement in RLS (32). Regarding QoL, conflicting evidence remains which gender benefits more (25, 27, 28, 32), while both genders show improvement in ADL (24–26, 29, 30, 33).

In general, possible gender mechanisms influencing movement disorders and their effect on STN-DBS could be explained by sexual dimorphism, genetics and hormones (70). Sexual dimorphism seems to be relevant in the neurological system (71). A meta-analysis examining sexual dimorphism in neural structure found that men generally have larger brain volume and exhibit differences in the volume and density of specific brain regions compared to women (72). Imaging studies show that PD leads to atrophy of the brain (73, 74). Interestingly, men with PD seem to show decreased cortical thickness in multiple brain regions compared to healthy controls, which suggests more cognitive decline in men with PD than women with PD (75). Furthermore, when investigating neural activity, in studies involving healthy individuals, it has been reported that women exhibit higher beta power compared to men (76, 77). In addition, in PD patients undergoing STN-DBS, gender differences in neural activity have been observed (78). Compared to men, women with PD displayed higher power in the alpha/low-beta bands OFF medication, as well as higher gamma and 300 Hz rhythm bands ON medication (78). These findings suggest that there are gender differences in the general anatomical structure and neural activity, which may potentially contribute to different responses to DBS that aims to normalise neural activity. There seem to be some gender differences within genetics in PD (70). X and Y chromosomes seem to individually influence the pathogenesis of PD (79), which could lead to a difference between genders. When looking specifically into genes, it seems that there are differences in the expression and functioning of dopaminergic and substantia nigra genes between genders, which could contribute to gender differences in PD presentation and response to treatment (80, 81). The hormonal environment of women differs from men, which could explain gender differences. Sex hormones affect the brain, which contribute to gender differences in neural function (82). This seems to be the in particularly the case for oestrogen (82). Oestrogen has a pro-dopaminergic effect, independent of oestrogen receptors, which protects against the pathogenesis of PD (79, 83). On the other hand, androgens do not exert a similar neuroprotective effect (79). These gender differences in hormones could possibly contribute to the gender-specific effects of STN-DBS.

Lastly, the psychological perception and social environment of women differ from men. Women are known to endure more emotional disorders, such as depression (84, 85). There seems to be a role for female-specific factors, such as coping and reporting, influencing the disease presentation (85). Their different emotional perception could contribute to a difference in perceived effect, especially for subjective measures, such as questionnaires. Furthermore, women often have a major role in their household and corresponding chores (86), which could possibly influence the rate and perception of recovery, after STN-DBS.

Future research should focus directly on the study of gender differences. By focusing specifically on gender outcomes in a prospective setting, rather than reporting them as a secondary outcome, potential problems with multiple testing can be avoided. In addition, more studies should include non-surgical control groups and directly compare gender groups rather than simply reporting differences within each gender group before and after surgery. These approaches will contribute to a more accurate and comprehensive understanding of sex differences in PD patients treated with STN-DBS.

In summary, there appears to be evidence of gender discrepancies and gender differences in clinical outcomes in PD patients treated with STN-DBS. These findings are partially consistent with gender differences in the general PD population. High quality future research focusing specifically on gender differences in PD patients treated with STN-DBS is needed to better understand gender differences in STN-DBS PD patients. This may have important clinical implications for patient selection, outcome prediction and management of PD patients treated with STN-DBS.

## Data availability statement

The original contributions presented in the study are included in the article/Supplementary material, further inquiries can be directed to the corresponding author.

## Author contributions

MH: Data curation, Formal analysis, Investigation, Project administration, Visualization, Writing – original draft, Writing – review & editing. RV: Funding acquisition, Supervision, Validation, Writing – review & editing. DG: Conceptualization, Formal analysis, Funding acquisition, Investigation, Methodology, Project administration, Resources, Supervision, Validation, Writing – review & editing.
