# Supplementary material for: Gender discrepancies and differences in motor and non-motor symptoms, cognition, and psychological outcomes in the treatment of Parkinson’s disease with subthalamic deep brain stimulation
Source: Front Neurol. 2024 Jan 8;14:1257781. doi: 10.3389/fneur.2023.1257781 (PMC10800523; doi:10.3389/fneur.2023.1257781)
Supplement: Supplementary file 1 [file Data_Sheet_1.DOCX]

**Supplementary material.** Search strings used to select relevant papers for the review.

**Pubmed**

("Parkinson Disease"[Mesh] OR "Parkinson's"[Tiab] OR "Parkinson"[Tiab] OR "PD"[Tiab]) AND ("Subthalamic Nucleus"[Mesh] OR "Subthalamic"[Tiab] OR "Deep Brain Stimulation"[Mesh] OR "DBS"[Tiab] OR "deep brain stimulation"[Tiab] OR "STN"[Tiab]) AND ("Sex Characteristics"[Mesh] OR "sex"[Tiab] OR "gender"[Tiab] OR "genders"[Tiab] OR "sexes"[Tiab] OR "males"[Tiab] OR "females"[Tiab] OR "men"[Tiab] OR "women"[Tiab])

**Web of Science**

TS=("Parkinson's" OR "Parkinson" OR "PD") AND TS=("Subthalamic" OR "Deep Brain Stimulation" OR "DBS" OR "STN") AND TS=("Sex Characteristics" OR "sex" OR "gender" OR "genders" OR "sexes" OR "males" OR "females" OR "men" OR "women")
